# Disentangling the coexistence strategies of mud-daubing wasp species through trophic analysis in oases of Baja California peninsula

**DOI:** 10.1371/journal.pone.0225266

**Published:** 2019-11-21

**Authors:** Armando Falcón-Brindis, Ricardo Rodríguez-Estrella, María Luisa Jiménez

**Affiliations:** Entomology and Arachnology Laboratory (Laboratorio de Aracnología y Entomología), Conservation and Environmental Planning Program (Programa de Planeación Ambiental y Conservación), Northwest Biological Research Center (Centro de Investigaciones Biológicas del Noroeste), La Paz, Baja California Sur, México; Universidade Federal de Uberlândia, BRAZIL

## Abstract

Species within the same trophic level show different strategies to avoid competition. Among these mechanisms, differences in body size, spatio-temporal segregation, and diet preference often leads to a niche partitioning. Nonetheless, little attention on coexisting predatory insects and their network interactions has been paid. In this study, we analyzed the strategies to avoid competition among three sympatric mud-daubing wasps of the genus *Trypoxylon* (Hymenoptera: Crabronidae) in oases and their surrounding xeric area from the Baja California peninsula, Mexico. We compared the prey richness, composition and proportion of spider guilds that were captured by the wasps. We tested whether the differences in wasp body size explained the niche breadth, niche overlap and the size of spider prey. We assessed the spider-wasp interactions through a network analysis. With the use of trap-nests, we collected 52 spider species captured by the wasps. Both the guild and species composition of preyed spiders was different between the three wasp species. Differential proportions in the capture of spider guilds and a little diet overlap were found among the wasp species. We found that the wasp body size was positively correlated with prey size, but it was not a *proxy* of niche breadth. Moreover, the largest wasp species was able to nest in both mesic and xeric habitats, while the two smaller species were restricted to the oases. This study reveals that the diversity of spiders in oases of Baja California peninsula is crucial to maintain highly specialized oasis-dependent wasp species. The niche partitioning between mud-daubing wasps can be shaped by their inherent body size limitations and hunting strategies through foraging specialization for specific spider guilds. Food selection and slight differences in body size reduce competition and allow the coexistence of sympatric wasps. Our study is the first approach exploring the interaction networks between mud-daubing wasps and their spider preys, highlighting new insights into the morphological and ecological factors that shape antagonistic interactions, and allow the coexistence of predators in deserts.

## Introduction

Among sympatric species, the competition and availability of resources determine how they coexist in the community [1, 2]. When species compete for the same food, space or any environmental resource, several mechanisms are displayed to avoid niche overlap [3], as well as constrains or expansions of niche breadth and dispersion abilities [4–6]. The body size is among morphological factors influencing the niche breadth [7–9], thus, in a cascade model of trophic levels, larger predators would eat larger prey [10, 11]. Additionally, since body size can represent physiological limitations to the foraging range, it can have a positive correlation with the home range [9] and food consumption [12]. However, the niche segregation can be more difficult to explain among sympatric species with similarities in body size (as in cryptic species), since they are expected to be ecologically analogues [13].

On the other hand, behavioral differences (e.g. foraging specialization, territoriality) can be strong mechanisms to avoid niche overlap [14]. Although predators often have diet shifts according to the availability of prey, specific patterns of searching strategies still prevail [15]. Among terrestrial predatory arthropods, food selection (i.e. prey size, species availability), hunting strategies, nesting behavior and breeding season have been pointed out as trade-offs for their coexistence [10]. It has been documented that nesting preferences, individual diet specialization and experience can be important factors to modify the hunting strategies among predatory wasps and are probably helping to reduce competition [16–19]. Female digger wasps can segregate their diets by patrolling different specific areas [20], or by hunting the most abundant prey during the breeding season [21]. In this sense, niche breadth can be strongly influenced by individual specialization and varies among species, populations, or even by the spatio-temporal context [22, 23].

**Fig 1:**
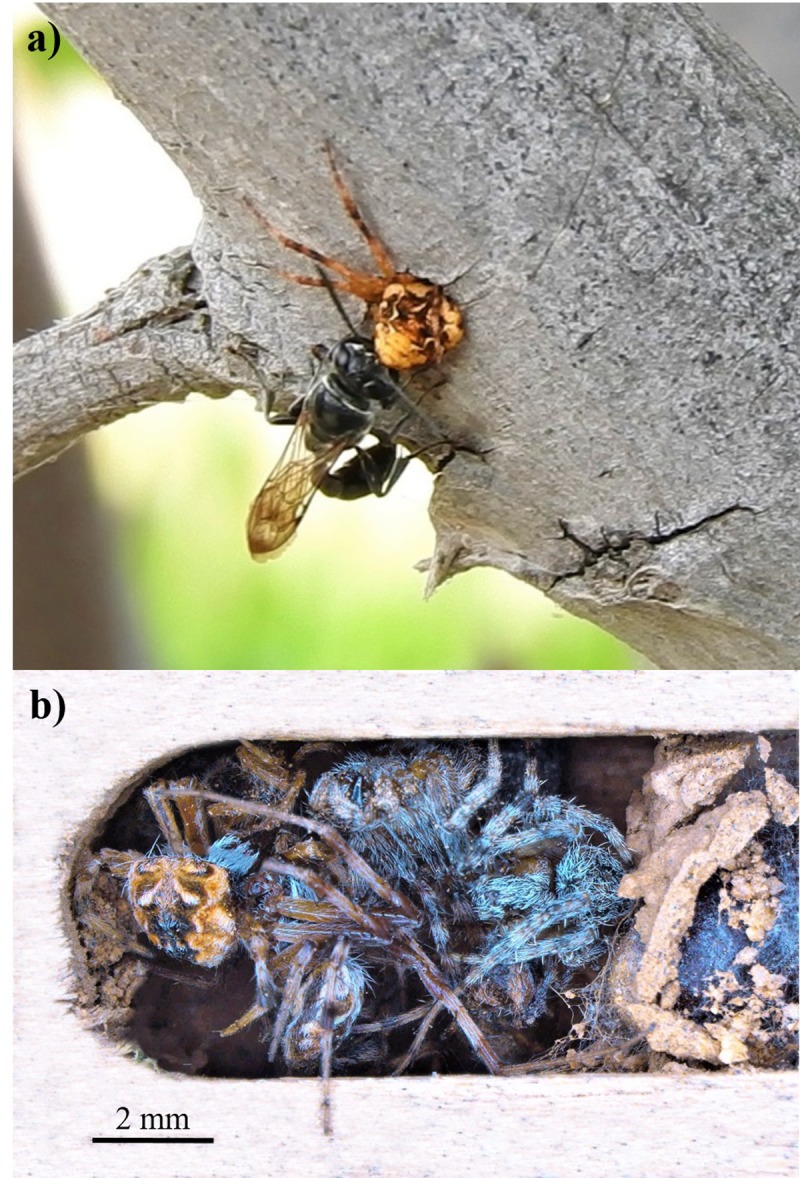
*Trypoxylon* wasp. **a)** Female wasp introducing a captured orb-weaving spider into her nest (i.e. an abandoned beetle bored stem). **b)** Brood cell provisioned with paralyzed spiders.

The behavioral and morphological factors shaping the niche breadth of highly sympatric species is still poorly understood, especially within the group of spider-hunting wasps. Those in the genus *Trypoxylon* Latreille (Hymenoptera: Crabronidae) are broadly known to feed their offspring with a variety of spider species [24–26] (Fig 1). Regardless the abundance of spiders in the community, most *Trypoxylon* wasps seem to be specialized for particular spider families [27], but the strategies to avoid competition remain unknown among sympatric wasp species. In this sense, ecological data of spider guilds may offer meaningful explanations to understand the foraging strategies of wasps and competition [28, 29].

In insular habitats, where niche overlap tends to be higher than in mainland [30], the rapid adaptation to environmental conditions plays a key role in the niche segregation of species [31]. Patchy habitats can offer insular-like conditions that harbor unique biological assemblages that coexist with limited resources [32]. Within this framework, oases are insular-like mesic habitats immersed in xeric ecosystems, with contrasting structure and function for several species. Such contrast is very important for conservation programs, especially since oases are small, fragile, and isolated environments [33, 34]. In these habitats, mechanisms to reduce competition between predatory arthropods are poorly understood, and the interactions with more than two-hundred spider species occurring in the oases from the Baja California peninsula are practically unknown [35]. In this study, we focused on disentangling those strategies that reduce the competition between *Trypoxylon* wasp species in oases from the Baja California peninsula to elucidate the strategies that allow their coexistence. Three *Trypoxylon* species were studied, *T. (Trypoxylon) bridwelli* Sandhouse, *T. (Trypargilum) dubium* Coville and *T. (Trypargilum) tridentatum tridentatum* Packard. Considering the isolation and limited food resources in oases from the Baja California peninsula [36], we hypothesized that the three *Trypoxylon* wasp species avoid food competition through a differential use of spider prey. Since these wasp species share a number of morphologically similarities, we also hypothesized that the wasp body size plays a role in the size of spider prey, with the prediction that wasp body size is positively correlated with prey size.

## Materials and methods

### Ethics statement

Field work was conducted with permission of Secretaría del Medio Ambiente y Recursos Naturales, in agreement with the Subsecretaría de Gestión para la Protección Ambiental and the Dirección General de Vida Silvestre who issued the Scientific Collector Permit: SGPA/DGVS/09769/15. Our study does not involve any endangered or protected species.

### Study area

Sampling was carried out from April to September during 2016 and 2017 on six localities along the Baja California peninsula (BCP), in northwest Mexico. The north portion of the BCP belongs to the Nearctic region, which is represented by xeric scrublands typical of the Sonoran desert. In the southernmost area, there are taxa with Neotropical affinity and vegetation such as tropical deciduous dry forest [37]. The sampled areas included oases located between 23°N and 30°N, with sizes ranging from 0.06 to 2.6 km^2^ in size (Table 1).

**Table 1:** Sampled localities along the Baja California peninsula. SA = Santiago, EP = El Pilar, LP = La Purísima, ES = El Sauzal, SB = San Borja, SF = San Fernando. Temperature (°C), precipitation (mm), and relative humidity (Rh) were obtained from SMN [38]. Size was extracted from maps from INEGI [39].

Oasis	Lat	Long	Size (km^2^)	Elev (m)	°C	mm	Rh (%)
SA	23°28’	109°43’	1.47	132	23.7	330.2	67.6
EP	24°28’	111°00’	0.25	120	22.2	154.8	65.6
LP	26°11’	112°04’	2.69	95	22.9	127	50.8
ES	27°10’	112°52	0.21	150	21.9	121.1	63.3
SB	28°44’	113°45’	0.06	445	19.9	114.1	42.1
SF	30°00’	115°14’	1.29	450	18.2	91.1	37.8

### Sampling protocol

We focused our analyses on three wasp species that occupied the nests (i.e. 133 in total): *Trypoxylon bridwelli* (31 nests), *T. dubium* (47) and *T. tridentatum* (55). We used artificial cavities to collect the wasp´s nests and identify their composition (i.e. spider prey). We set the traps in both the oases and the surrounding desert to evaluate the prey composition between habitats. The traps were set in gradual distances from the edge of the waterbody (inside the oasis) towards the desert area. That is, from around 5.0 m to 3,500 m away from the waterbody.

In total, we offered 2,430 cavities equally distributed in 162 trap nests set in the six localities. Trap nests consisted in three wooden blocks (10x17x2.5 cm each) set 1.5–2.0 m height, piled and gripped together. Each nest contained five rows of tunnels with different diameter (3.1, 6.3, 1.9, 9.5, 12.7 mm) x 150 mm long [25]. From April to September 2016 we sampled the southernmost oases: Santiago (SA), El Pilar (EP), and La Purísima (LP). From April to September 2017, the northernmost oases were sampled: El Sauzal (ES), San Borja (SB), and San Fernando (SF). In both years, we monthly replaced the occupied traps.

The morphological differences of adults, immature larvae and external appearance of pupae allowed the identification of wasp species and thus the association with their spider prey [26]. Inside the nests, the brood cells were carefully inspected, sorting and counting the spiders for taxonomical identification. Taxonomic identification was done in the laboratory of Arachnology and Entomology (CARCIB) of the Centro de Investigaciones Biológicas del Nororeste (CIBNOR). Since the great majority of spiders were juveniles, identification to species level was not always possible. However, the vast material of spiders from Baja California Sur deposited in the CARCIB, allowed the correct distinction of most morphospecies. Because in some cases we found the remaining parts of spider prey within the cells (i.e. already devoured by the wasp larva), the identification was possible to family level.

### Diversity and composition of prey

Spider prey was categorized into the trophic guilds suggested by Cardoso et al. [28] and Uetz et al. [40]. These guilds were proposed as surrogates of spider families based on their foraging strategy, prey range, and vertical stratification. Based on these categories, we classified the spiders captured by the *Trypoxylon* species within the following guilds: ambush hunter, orb web, spatial web, stalker, ground hunter, and specialist. For each wasp species, we calculated alpha diversity of prey using Hill numbers and estimated the number of spider species through Chao-1 [41, 42]. We compared the diversity of the overall spider prey captured by the wasps. The rarefaction curves for each wasp species were estimated. These analyses were computed on R v3.5.1 using the *iNEXT* package [43, 44].

To test the differences in composition of the spider communities captured by the three wasp species, we used a Permutational Multivariate Analysis of Variance (PERMANOVA) [45]. A multivariate analysis of homogeneity of group dispersions (PERMDISP) was used to test heterogeneity of the prey community and as a measure of beta diversity [46]. We used a Non-metric Multidimensional Scaling (NMDS) measured by Bray-Curtis distances to visualize the dissimilarities in the composition of spider species captured by the wasp species [47]. These analyses were done with *vegan* package in R [44, 48].

To evaluate if there is an effect of habitat and time in the amount of food the wasps can provide to the offspring, we used generalized linear mixed models (GLMM) to assess if the length of brood cells depends on the habitat (oasis or desert) or if it varied through the time (i.e. monthly variation). Models were fitted with *lme4* package [49] in R [44].

### Niche partitioning

In order to determine the relative level of dietary specialization and niche segregation of the species, the niche breadth and niche overlap were calculated for the three *Trypoxylon* wasps [50]. Hurlbert's formula [51] was applied to obtain the standardized niche breadth (Bj) [52]. We used the Pianka´s index to measure niche overlap. Niche breadth index ranges from 0 to 1, where values close to 1 indicate more specialization. Pianka´s index also ranges from 0 to 1, where values close to 1 indicate a higher diet overlap. We used the *spaa* package to compute these indexes [53]. We built a bipartite network (predator-prey) to calculate the complementary specialization index (H_2_´) and Shannon diversity of interactions between both trophic groups [54]. H_2_´ index ranges between 0 (no specialization) and 1 (complete specialization). Since this index can be sensitive to matrices constructed with few species, we compared our data against 1000 random null models that avoid biases regardless the matrix size. With the null model approach, we assessed that the spider-wasp interactions are not being reflections of the sampling properties, thus producing random links between predator and prey [55, 56]. The network analysis was done with the package *bipartite* in R [44, 57, 58].

To test whether the size of predators influenced the niche breadth and prey size selection, we measured morphological attributes of size on both wasps and spiders. We obtained the values of body length, facial and intertegular distance from the three *Trypoxylon* species. The spider size was represented with the values of cephalothorax´s length and width [59]. Only female wasps were considered since they are the responsible of hunting and transportation of spiders to the nests [24]. The correlation between the wasp size and cephalothorax´s length-width ratio of spiders was tested. We used a Linear Discriminant Analysis (LDA) to evaluate whether the three wasp species were well separated according to the morphological measurements [60]. LDA was calculated with the *psych* package in R [44, 61]. Measurements were done under a stereo microscope Nikon SMZ25, with 11x magnification, 1.1x zoom and a Nikon SRH 1x objective.

## Results

**Fig 2:**
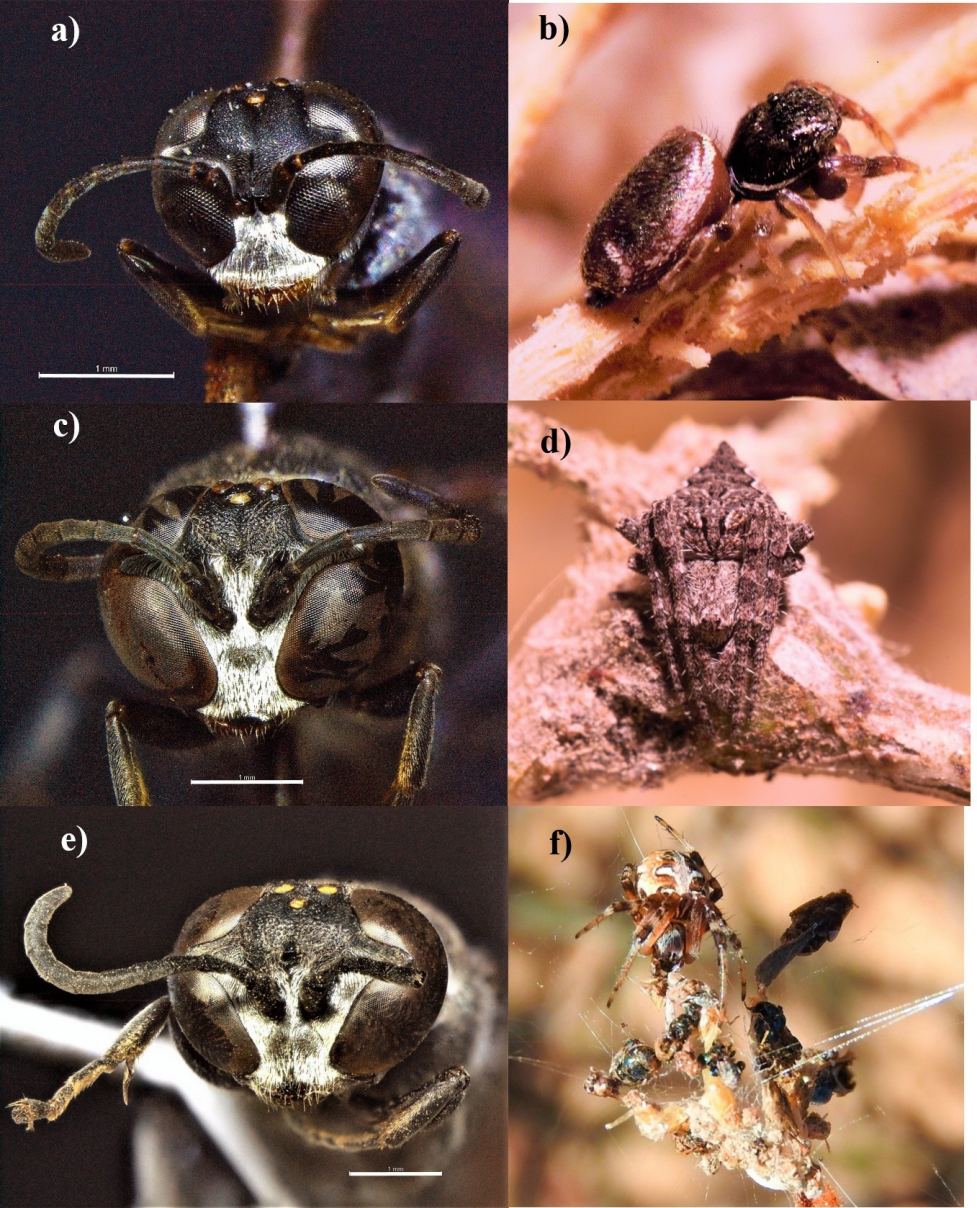
Studied *Trypoxylon* species and their most common preyed spiders. **a)** *T. bridwelli* and **b)** *Sassacus vitis*, **c)** *T. dubium* and **d)** *Eustala* sp., **e)** *T. tridentatum* and **f)** *Metepeira arizonica*. Photographs by A. Falcón-Brindis and Luis E. Robledo Ospina.

Altogether, 670 spiders were captured by the three *Trypoxylon* species that occupied 133 nests. The spider prey comprised 11 families, 28 genera and 52 species divided into six guilds (Table 2). Globally, a total of 45 spider species were preyed upon in oases and seven spider species in the desert. The occurrence of spider species varied between the wasp species, but the most common spiders included *Sassacus*, *Eustala* and *Metepeira* (Fig 2). *Phidippus phoenix* Edwards (Salticidae) was the unique spider prey captured by the three wasp species. Individuals of the family Salticidae were the most recurrent in the nests (38.3%), followed by Araneidae (33.2%) and Theridiidae (17.3%). *Sassacus vitis* Cockerell (Salticidae) (27.2%), *M. arizonica* (Araneidae) (22.8%), and *Theridion submissum* Gertsch & Davis (Theridiidae) (16.6%) were the most frequent species in the nests. Only *Trypoxylon bridwelli* captured two different species of ant-mimic jumping spiders (*Peckhamia* spp.), while only *T. dubium* and *T. tridentatum* included nocturnal spiders in the diet (Table 2).

**Table 2:** List of spider species and their guild category captured by each mud-daubing wasp. AH = ambush hunter, OW = orb web, SW = spatial web, ST = stalker, GH = ground hunter, SP = specialist. Spiders within the nest in oasis = ●, desert = ○, and both habitats = Δ. Guild categories from Cardoso et al. [33] and Uetz et al. [46].

			Wasp species		
Family	Spider species	Guild	*T. bridwelli*	*T. dubium*	*T. tridentatum*
Anyphaenidae	*Hibana incursa*	ST		●	
	*Hibana sp*.	ST			●
Araneidae	*Cyclosa turbinata*	OW			○
	*Eustala californiensis*	OW			●
	*Eustala sp*.	OW		●	●
	*Gen. sp1*	OW		●	
	*Gen. sp2*	OW			●
	*Metepeira arizonica*	OW			Δ
	*Metepeira crassipes*	OW		●	
	*Meteperia sp*.	OW			●
	*Neoscona sp1*	OW		●	○
	*Neoscona sp2*	OW			●
	*Larinia sp1*	OW			●
Dictynidae	*Emblyna sp*.	SW	●		
	*Dictyna sp1*	SW		●	
	*Dictyna sp2*	SW			●
	*Mallos pallidus*	SW		●	
Cheiracanthiidae	*Cheiracanthium sp*.	ST		●	
Gnaphosidae	*Gen. sp*	GH			●
Mimetidae	*Mimetus sp*.	SP			●
Oxyopidae	*Hamataliwa sp*.	ST	●		
	*Oxyopes flavus*	ST		●	
	*Oxyopes salticus*	ST		●	●
Philodromidae	*Tibellus sp*.	AH		●	●
	*Gen. sp*.	AH			●
Salticidae	*Colonus sp*	ST	△		Δ
	*Gen. sp1*	ST		●	
	*Gen. sp2*	ST		●	
	*Gen. sp3*	ST	●		
	*Habronattus ammophilus*	ST		●	
	*Habronattus californicus*	ST	●		
	*Habronattus conjunctus*	ST	●		
	*Habronattus pyrrithrix*	ST	●		
	*Habronattus sp*.	ST	●		
	*Marpissa robusta*	ST			●
	*Paramarpissa sp*.	ST	●		
	*Peckhamia picata*	SP	●		
	*Peckhamia sp*.	SP	●		
	*Phidippus phoenix*	ST	●	●	●
	*Salticus sp*.	ST	●		
	*Sarinda cutleri*	ST	●		
	*Sassacus vitis*	ST	●		
	*Sassacus sp*.	ST	●		●
Theridiidae	*Euryopis sp*.	SW	●	●	
	*Gen. sp*	SW		●	
	*Latrodectus hesperus*	SW			○
	*Theridion sp*.	SW		●	
	*Theridion submissum*	SW			○
Thomisidae	*Mecaphesa celer*	AH		●	
	*Mecaphesa sp*.	AH	●	●	
	*Xysticus sp*.	AH		●	
	*Xysticus phoenix*	AH	●		

Nocturnal spider species are shaded grey.

**Fig 3:**
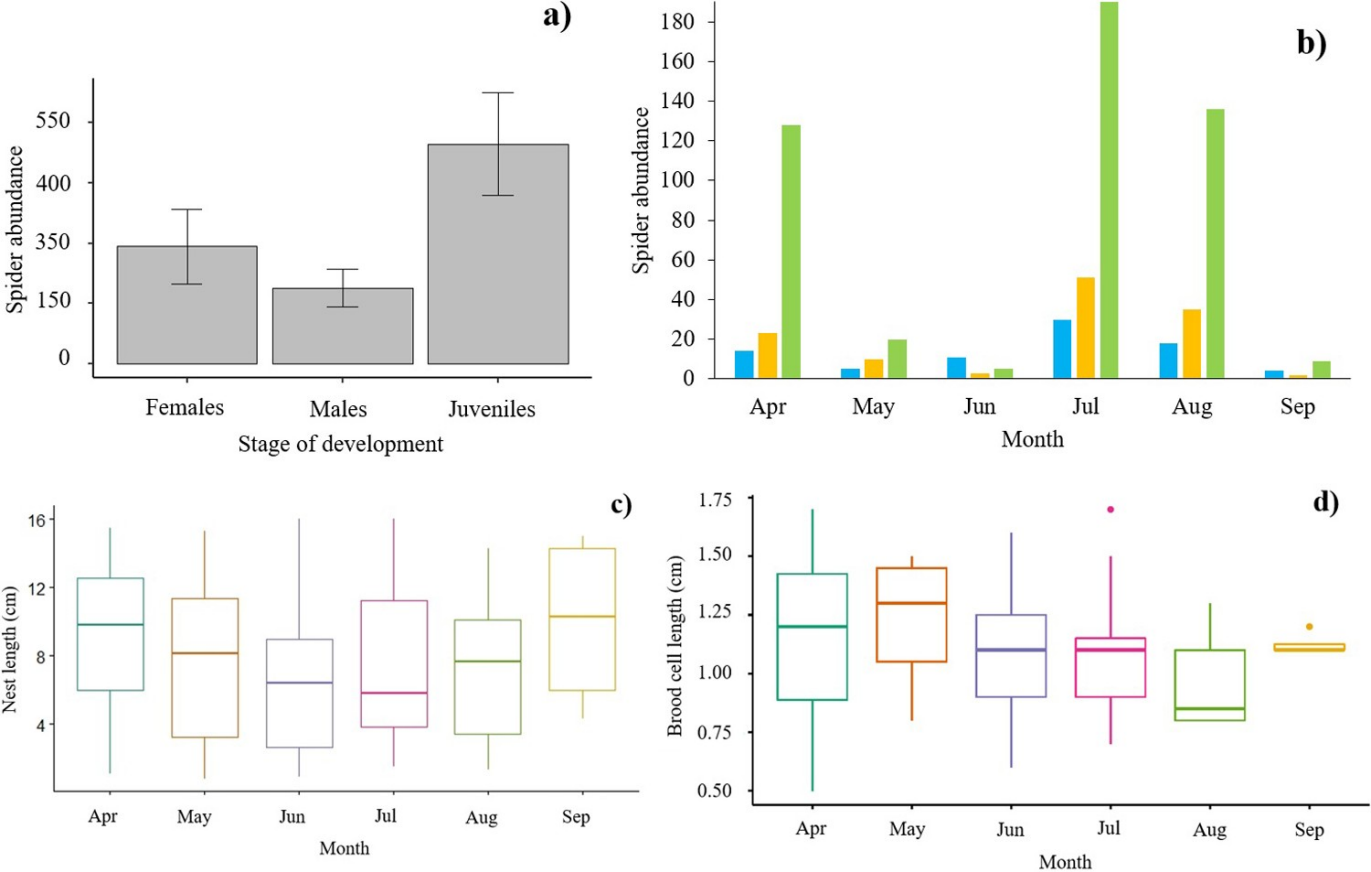
Spiders captured by the *Trypoxylon* wasps throughout the localities. **a)** Life cycle stage of captured spiders; abundance was counted as adults (male, female) and juvenile individuals; **b)** monthly variation of total spider abundance **c)** nest length and **d)** cell length. Whiskers represent the standard error. Blue bars = males, orange = females, green = juveniles.

The abundance of spiders in the diet varied accordingly to the stage of development during the life cycle (H = 33.8, d.f. = 2, *p <0.001*). The vast majority were in juvenile stages (69%) (Fig 3A). The peak of prey abundance occurred in July (i.e. 43.6%) (Fig 3B). However, neither the habitat (GLMM, χ^2^_1_ = 2.56, *p* = 0.11) nor the month of the year were predictors of the length of brood cells (χ^2^_5_ = 7.46, *p* = 0.18) and nest length (χ^2^_5_ = 2.64, *p* = 0.75) (Fig 3C and 3D). The temporal pattern of abundance across the time was the same either for males (H = 2.2, d.f. = 3, *p =* 0.531), females (H = 1.6, d.f. = 3, *p =* 0.657) and juvenile spiders (H = 2.22, d.f. = 3, *p =* 0.527).

**Fig 4:**
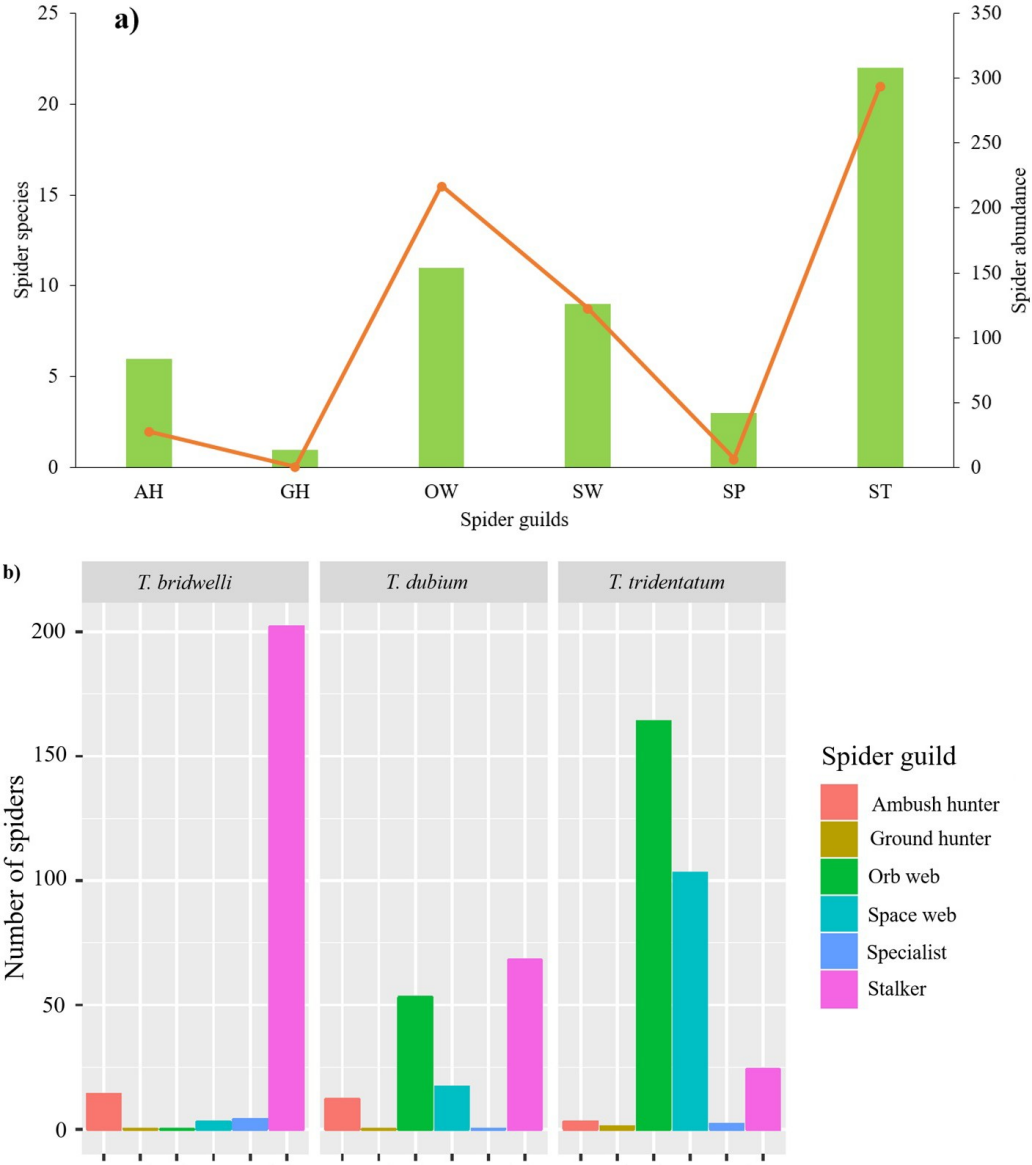
Captured spider guilds. **a)** Total species richness and abundance per spider guild. Bars represent the richness (q = 0) and the line the abundance (right axis). **b)** Abundance of spider guilds in the diet of each wasp species. AH = ambush hunter, OW = orb web, SW = spatial web, ST = stalker, GH = ground hunter, SP = specialist.

Among the six spider guilds, the group of stalkers was the most abundant (43.8%) and had the highest richness (42.3%) within the nests (Fig 4A). The number of spider species per guild varied among wasps (χ^2^_10_ = 20.6, *p* = 0.024). None of the wasp species included all spider guilds in their diet. *Trypoxylon bridwelli* consumed mostly stalkers (68.4%), *T. tridentatum* mainly orb weavers (40.9%), and *T. dubium* was less selective, combining prey from stalkers (38%) and space web guilds (28.5%) (Fig 4B).

**Fig 5:**
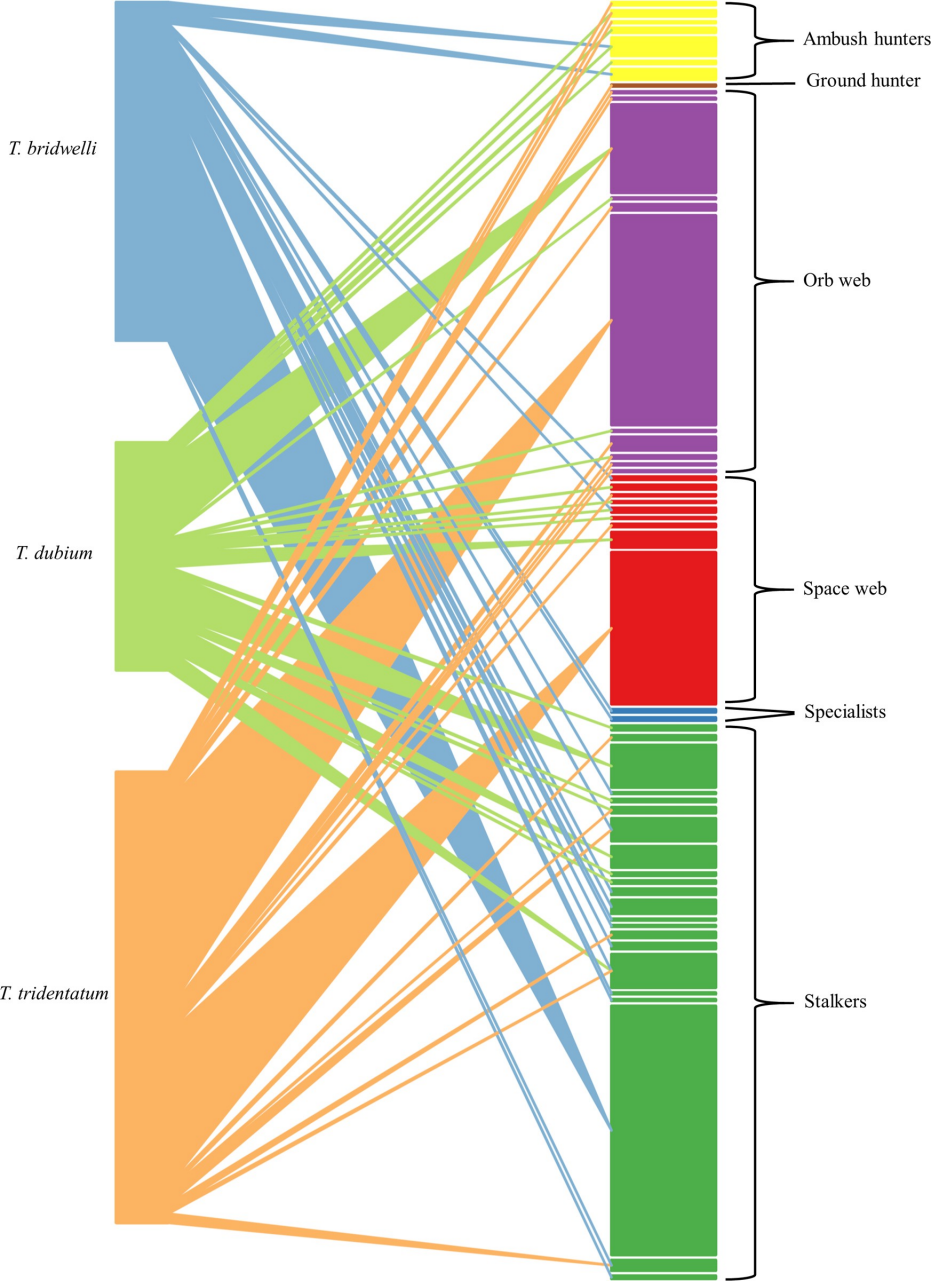
Bipartite interaction between wasps and spider guilds. Each guild contains the total number of spider species (right bars) preyed upon by each wasp species (left bars). The thickness of bars indicate the abundance of predators and prey, respectively. The line thickness represents the abundance of individuals captured by each wasp species.

The bipartite interactions between prey and predators showed a high degree of both overall specialization (H_2_´ = 0.89) and diversity of interactions (H´ = 2.6). Our network significantly differed from expected null models (Mean ± S.D.: 0.09 ± 0.01, *p*<0.001) (Fig 5).

**Fig 6:**
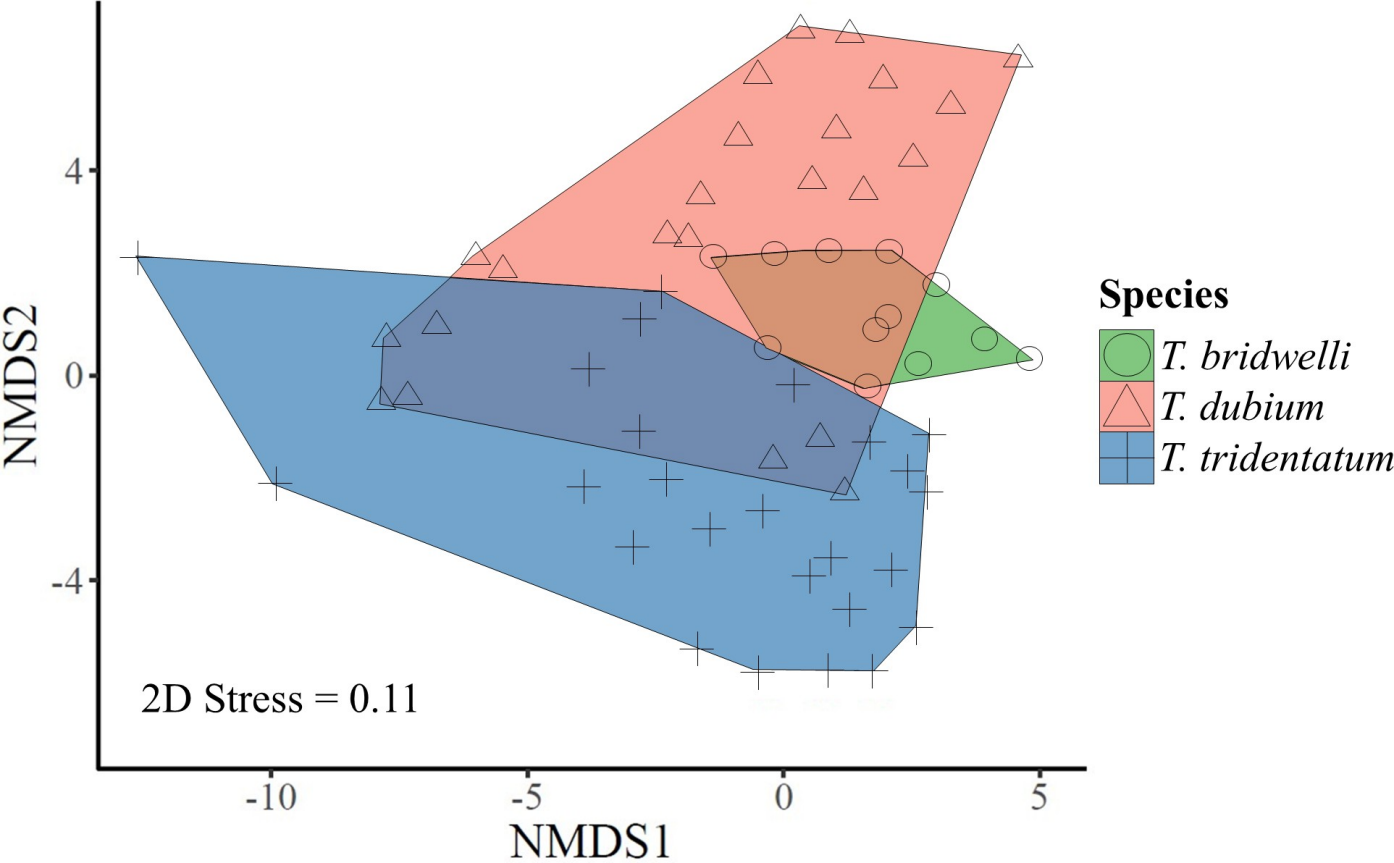
Non-metric multidimensional scaling of the spider prey composition. See the high dissimilarity between wasp species.

The community structure of spider prey was significantly different between the mud-daubing wasps (PERMANOVA *F* = 2.63, d.f. = 2, *p* = 0.001), and the variability in community structure (i.e. spider composition) was different between all of the wasp species (PERMDISP: F *=* 4.19, d.f. = 2, *p = 0.02*) (Fig 6).

**Fig 7:**
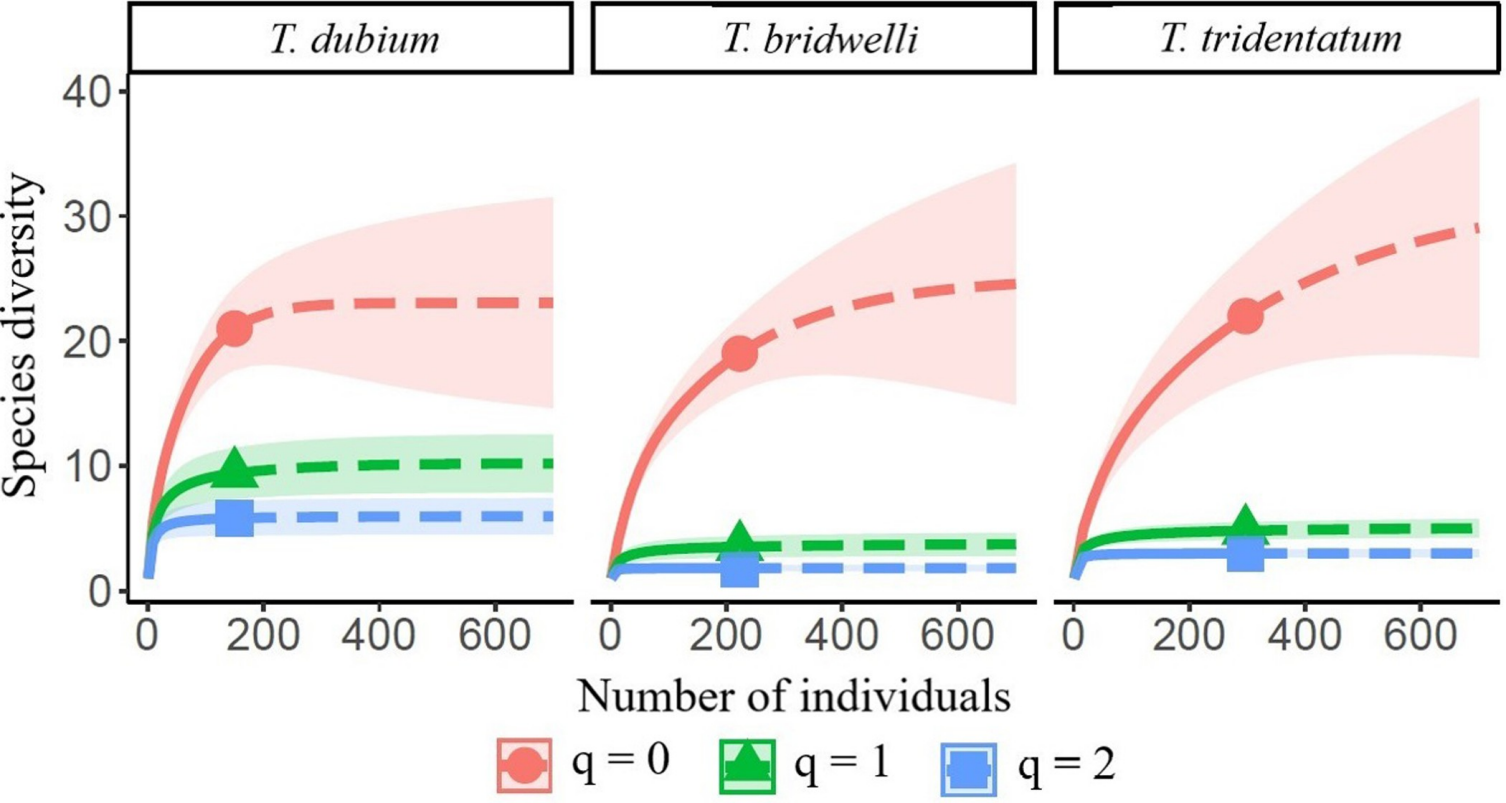
Species accumulation curve of captured spiders among the wasp species. The abscissa values represent the number of spiders that occurred within the wasps´ nests. The curves were extrapolated with 95% confidence intervals. Values of Hill numbers of order q = 0 (species richness), q = 1 (effective number of common species) and q = 2 (effective number of dominant species) are shown for each wasp species.

The spider prey diversity (H´) was significantly different between the three wasp species and the accumulation curves of prey diversity was asymptotic in *T. dubium* (Fig 7). *Trypoxylon tridentatum* showed the richest composition of prey and nocturnal species, whilst *T. dubium* showed the highest values of diversity, evenness and niche breadth. Moreover, *T. bridwelli* (the smallest species) had the highest dominant prey composition and the poorest species richness (Table 3). Diet overlap and similarity of prey species were very low among the three species. The highest values of both indices were found between the two largest wasps, *T. dubium* and *T. tridentatum* (Table 4).

**Table 3:** Summary of diversity and functional parameters between the three wasp species and their prey. The habitat indicates where the nests of each wasp species were found. The wasp body length is the measurement from the frons to the last segment of metasoma (only females). All estimators and diversity indices were calculated for the total number of spider species each wasp captured.

	Wasp species		
	*T. bridwelli*	*T. dubium*	*T. tridentatum*
Habitat use	oasis	oasis	oasis-desert
Wasp body length	9.7 ± 0.8 mm (n = 12)	11.7 ± 1.1 mm (n = 14)	13.2 ± 1.4 mm (n = 15)
Prey richness (*S*)	19	21	22
Prey evenness (*J*)	0.42	0.73	0.48
Chao-1	25.1	23.1	32.1
Prey diversity (H´)	1.26	2.24	1.48
Hill number (q = 1)	3.53	9.4	4.43
Prey dominance (D)	0.54	0.17	0.33
Nocturnal spiders	0	3	6
Niche breadth (Bj)	0.045	0.24	0.095

**Table 4:** Pairwise comparison of niche overlap using Pianka´s measure. Values of quantitative Sorensen index are in bold.

	*T. bridwelli*	*T. dubium*	*T. tridentatum*
*T. bridwelli*	-	0.011	0.002
*T. dubium*	**0.062**	-	0.041
*T. tridentatum*	**0.038**	**0.064**	-

**Fig 8:**
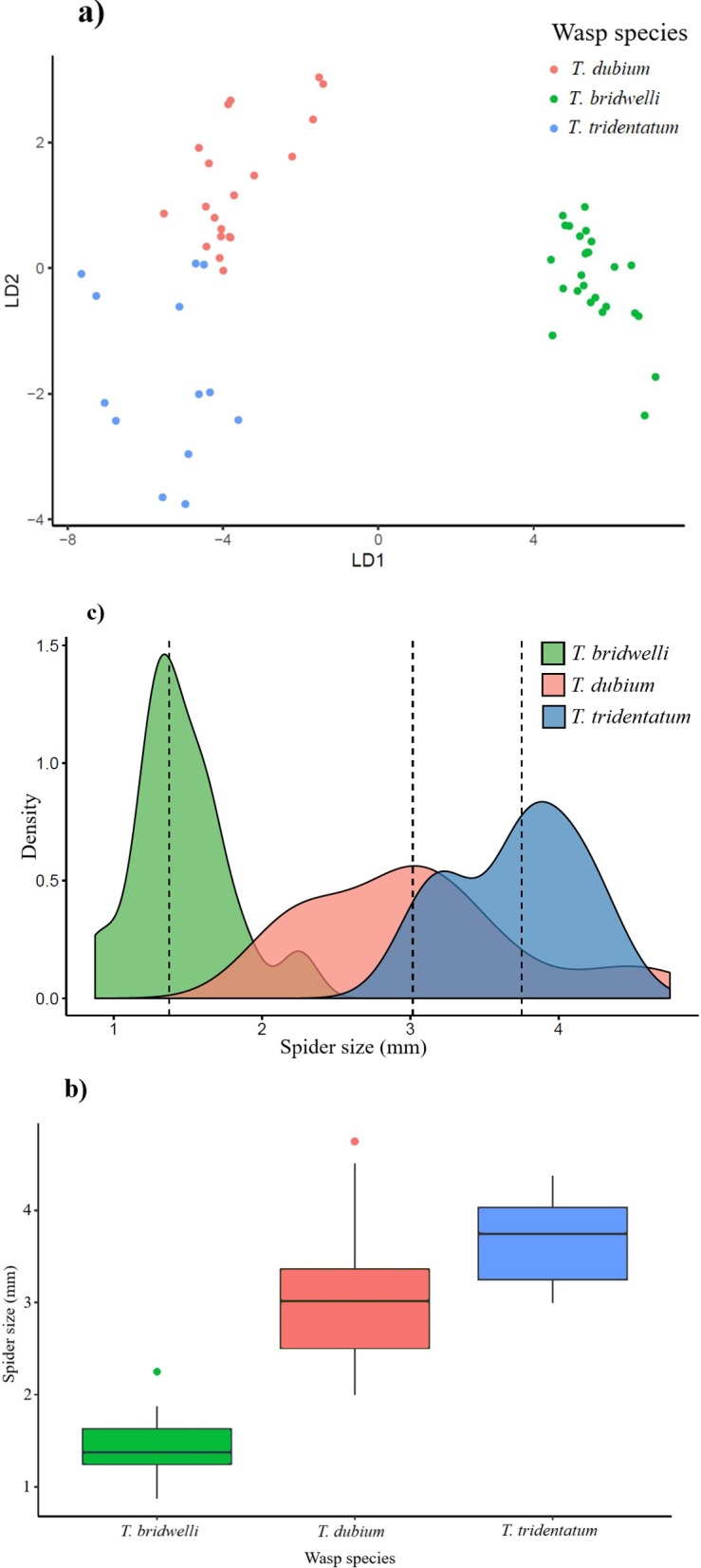
Morphological differences associated to prey selection. **a)** Differences in body size between the three wasp species, **b)** differences in spider prey size and **c)** range of prey size chosen by each *Trypoxylon* species. Dashed lines indicate the median.

There was a significant difference in the wasp body size that allowed the separation of the three *Trypoxylon* species (*F*_*2*, *69*_ *=* 773.6, *p<0.001*) (Fig 8A). Likewise, a significant difference in the selection of prey size was found between wasp species (*F*_*2*, *69*_ *=* 123, *p<0.001*) (Fig 8B). The wasp body size was positively and highly correlated with the spider size (*r* = 0.86, *p <0.001*), having *T. dubium* the widest range of prey size on its diet (2.0 to 4.75 mm length of cephalothorax) (Fig 8C).

## Discussion

According to our proposed hypotheses, each wasp species has a differential composition of spider prey. Therefore, there is a low diet overlap between wasps. Moreover, we confirmed that slight changes in the wasp body size (± 2 mm) play a role in the size of spider prey they hunt. As it was predicted, larger wasp species captured larger prey.

Usually, isolated habitats tend to harbor less species that display more specialized interaction networks [30]. As we found in this study, the wasp-spider interactions showed a highly specialized network, where the wasps have a strong prey preference. The use of resources in islands can be the result of selective pressures, since organisms undergo similar environmental pressures and dispersal limitations [30, 31]. Such pressures can also be found in isolated patchy environments (e.g. oases, caves, or sky islands), where coexisting species have developed several mechanisms to avoid competition [3, 32].

Although niche specialization can be high in insular habitats due to the restricted amount of resources [62], species can coexist through the niche segregation into multidimensional axes such as space, time, strata, or diet [37]. The three wasp species coexisting in the oases of Baja California Peninsula are displaying differential food specialization, which is probable the result of isolation processes that have led to evolutionary selective pressures.

*Trypoxylon* (*Trypargilum*) species within the Nitidum Species Group seem to be specialized for orb-weavers (Araneidae) and space web (Theriididae) species within the genera *Eustala*, *Metepeira*, *Eriophora* (Araneidae), and *Theridion* (Theriidiae) [26]. Similarly, we found that most of the diet of *T. tridentatum* and *T. dubium* (both within the Nitidum group) included araneid and theridid spider species. However, it is likely that both species richness and abundance of local prey at oases could have influenced the wasp´s prey choice. For example, the prey composition of *T. tridentatum* seems to be variable across localities: *Theridion submissum* (25% of total prey) in the southern Baja California peninsula [63, 64] and *Eustala rosae* (21%) in Arizona [25]. In our work, we found that *Metepeira arizonica* (46%) and *T. submissum* (33%) where the main target. Our results reinforce the idea that local composition of spiders may affect the wasp´s prey selection. Therefore, it is likely that *T. dubium* and *T. bridwelli* show a similar response, especially among oases from the BCP, where the spider composition is highly variable [36, 65].

### Selection of spider prey

It has been proposed that three-dimensional (3-D) webs are more efficient than two-dimensional (2-D) web architectures to avoid predation from mud-daubing wasps [27]. In some cases, 2-D weavers such as *Metepeira* species can build 3-D barrier webs [66]. However, whilst 2-D species rest at the center of webs, 3D-weavers remain protected inside the silk network. This defensive mechanism may explain why *Trypoxylon* are more prone to catch orb-weaving species. Although, spiders use other strategies to avoid predators (e.g. cryptic of coloration, silk retreats near webs, dropping from webs), web architecture is an important strategy of weaver spiders [67]. Even though the three wasp species preyed on web-building species, the specialization of *T. bridwelli* upon jumping spiders is noteworthy (>90% of prey species).

It has been suggested that both visual and chemical cues are likely the main mechanisms to locate either web-building or non-web-building species [68–72]. In our study, the inclusion of ant-mimic spiders in the diet of *T. bridwelli*, convey to the idea that probably chemical signals are being followed to locate its prey. Moreover, since spiders occupy different microhabitats and strata [73], it is likely that wasps are displaying specific patrolling behavior to locate their prey. In this sense, stratified sampling of spiders may help to understand the patterns of wasps´ foraging. More research is needed to determine the intricate combinations of chemical, aggressive mimicry, shape, and color perception involved during the spider recognition [74].

The nutritional composition of prey also plays a role in the food preferences of predators [75]. Among wasps, nutrition of larvae has a strong effect on reproductive fitness, foraging, and brain gene expression of adults [76, 77]. It makes conceivable that protein, lipid and carbohydrate supply is likewise critical in the fitness of adult *Trypoxylon* species. Especially because their offspring need to storage energy and protein before the diapause period [24]. Female *Trypoxylon* wasps usually prevent nutritional unbalance of larvae by providing more small body size juvenile spiders until the biomass reaches the required energy [78]. This is perhaps the explanation of the large number of juvenile spiders within the cells. However, the high amount of juvenile prey may be also an artifact of population dynamics of spiders or environmental effects on local the abundance [79].

### Effect of body size on prey preference

Since larger *Trypoxylon* wasps hunted larger spider prey, the effect of body size fits well with the assumption of cascade model (a positive predator-prey correlation) [10, 11]. However, this model is not always true for arthropods in particular assemblages [80], and the small amount of experiments is still a limitation to further assumptions. In our results, the body size was positively correlated with niche overlap but was not with the niche breadth. In this case, the overlap between wasps increased with the body size but the mid-sized species (*T. dubium*) showed the widest niche breadth and range of prey size. Similarly, Polidori et al. [81] showed that the body size between crabronid wasps was positively correlated with the prey size, but with the niche breadth. These authors suggest that most solitary wasps experience a strong individual specialization, which broadly influences the prey size selection. In addition, according to the flight muscle ratio, thus the load-lifting capability, only a few wasp species are optimal foragers, which is attributed to several biotic factors [82].

On the other hand, the seasonal variation can be a factor shaping the range of prey size and niche breadth of wasps. In northern Brazil, Araújo & Gonzaga reported variation in the prey selection of *Trypoxylon (Trypargilum) albonigrum*, finding a broader range of prey size and niche breadth during the wet season [83]. However, the relationship between the wasp, prey size and niche breadth is still under debate, especially since the prey preference of several wasp species remain unknown. Although the identification of spider prey could be challenging considering the large amount of juveniles captured by the wasps, the appropriate identification of morphospecies did not alter the analyses. In this sense, DNA approximations would be an option to avoid misleads in the prey identification.

### Habitat effect on niche partitioning

An alternative explanation for niche partitioning could be the effect of habitat use among wasp species [25]. In other *Trypoxylon* species, habitat is an important factor explaining the composition and abundance of spider prey [84, 85]. In our study, the highest abundance and richness of spiders were found in oases. The largest wasp species (*T. tridentatum*) used both oases and desert habitats and in general hunted larger spider prey. Among insects, physiological limitations have implications on their distribution and habitat use [86]. In a broad sense, insects with larger body size are able to tolerate more environmental variability [87]. Moreover, flying insects with larger body mass tolerate higher temperatures in extreme habitats (e.g. deserts) due to higher surface area-volume ratios [88]. In dry environments, water loss rates are lower in large bee species thus being less vulnerable to desiccation [89]. Although, temperature could explain the microhabitat selection of some bees and wasps [90, 91], the thermoregulatory physiology of wasps is still poorly understood [92]. Nonetheless, it seems that the body size of *T. dubium* and *T. bridwelli* may be a physiological boundary in both thermal tolerance and prey-lifting capacity.

Beyond microclimatic preferences, the coexistence of wasps could be mediated by temporal segregation. For example, in Northeast Brazil, Santos & Presley [93] observed that social vespid wasps had slight changes in their peak activity across the day, suggesting that environmental factors such as temperature and humidity are playing a role in the foraging activity, thus, reducing the interspecific competition. In other cases, the coexistence of social wasps is allowed by broad differences in their diet (i.e. specialist vs opportunistic species) [94].

## Conclusions

With these results, we disentangled the strategies that three mud-daubing wasp species have to coexist in isolated and small mesic environments into a desert ecosystem. Differential use of resources, either food or habitat (oasis or desert) allows the coexistence of ecologically similar wasp species. In this regard, each wasp species is showing a high guild preference, presenting a specialized predator-prey network to hunt certain spider species. Moreover, the wasp body size is positively correlated with prey size, allowing the ecological differentiation for the three *Trypoxylon* species.

On the other hand, the oases seem to work as islands for the studied wasp species, either as the product of physiological boundaries or food preference. Therefore, the conservation of isolated habitats become crucial for many species that depend on specific and limited resources. Moreover, habitat characteristics can be crucial for cavity-nesting species [95], but the direct effects on these guilds are poorly known. Since anthropogenic pressure is the main cause of disturbance in oases of Baja California [29], highly specialized sympatric insects could be threatened if habitat loss and fragmentation modify the structure of these insular-like environments.

## References

[1] Cornell H. Niche overlap In A Hastings Gross LJ, editors. Encyclopedia of Theoretical Ecology. California: University of California Press; 2012 p. 489–498.

[2] Clewlow HL, Takahashi A, Watanabe S, Votier SC, Downie R, Ratcliffe N. Niche partitioning of sympatric penguins by leapfrog foraging appears to be resilient to climate change. J Anim Ecol. 2018; 00:1–13. 10.1111/1365-2656.12919 30378103 PMC7379715

[3] MacArthur RH, Levins R. The limiting similarity, convergence, and divergence of coexisting species. Am Nat. 1967; 101: 377–385.

[4] Bolnick DI. Intraspecific competition favours niche width expansion in *Drosophila melanogaster*. Nature. 2001; 410: 463–466. 10.1038/35068555 11260712

[5] Yu DW, Wilson HB, Frederickson ME, Palominos W, De La Colina R, Edwars DP, Balareso AA. Experimental demonstration of species coexistence enabled by dispersal limitation. J Anim Ecol. 2004; 73: 1102–1114. 10.1111/j.0021-8790.2004.00877.x

[6] Sexton JP, Montiel J, Shay JE, Stephens MR, Slatyer RA. Evolution of ecological niche breadth. Annu Rev Ecol Evol Syst. 2017; 48: 183–203. 10.1146/annurev-ecolsys-110316-023003

[7] Churchfield S, Nesterenko VA, Shvarts EA. Food niche overlap and ecological separation amongst six species of coexisting forest shrews (Insectivora: Soricidae) in the Russian Far East. J Zool. 1999; 248: 349–359.

[8] Woodward G, Hildrew AG. Body-size determinants of niche overlap and intraguild predation within a complex food web. J Anim Ecol. 2002; 71: 1063–1074. 10.1046/j.1365-2656.2002.00669.x

[9] Jeglinski JWE, Goetz KT, Werner C, Costa DP, Trillmich F. Same size–same niche? Foraging niche separation between sympatric juvenile Galapagos sea lions and adult Galapagos fur seals. J Anim Ecol. 2013; 82: 694–706. 10.1111/1365-2656.12019 23351022

[10] Wilson DS. The adequacy of body size as a niche difference. Am Nat. 1975; 109: 769–784.

[11] Cohen JE, Pimm SL, Yodzis P, Saldaña J. Body sizes of animal predators and animal prey in food webs. J Anim Ecol, 1993; 62: 67–78.

[12] Saulinho HHL, Trivinho-Strixino S. Body length determines the diet and niche specialization of non-biting midge predator (Tanypodinae) larvae in shallow reservoirs. Neotrop Entomol. 2019; 48: 136–142 10.1007/s13744-018-0620-9 30039478

[13] Scriven JJ, Whitehorn PR, Goulson D, Tinsley MC. Niche partitioning in a sympatric cryptic species complex. Ecol Evol. 2016; 6: 1328–1339. 10.1002/ece3.1965 26848386 PMC4730923

[14] Begon M, Townsend CR, Harper JL. Ecology, from individuals to ecosystems. Oxford: Balckwell Publishing; 2006.

[15] Cornell H. Search strategies and the adaptive significance of switching in some general predators. Am Nat. 1976; 110: 317–320.

[16] Punzo F. Experience affects hunting behavior of the wasp, Pepsis Mildei Stål (Hymenoptera: Pompilidae). J N Y Entomol Soc. 2005; 113: 222–229. 10.1664/0028-7199(2005)113[0222:EAHBOT]2.0.CO;2

[17] Evans HE, O’Neill KM. The sand wasps: natural history and behavior. Cambridge: Harvard University Press; 2007.

[18] Araújo MS, Gonzaga MO. Individual specialization in the hunting wasp Trypoxylon (*Trypargylum*) albonigrum (Hymenoptra, Crabronidae). Behav Ecol Sociobiol. 2007; 61: 1855–1863. 10.1007/s00265-007-0425-z

[19] Pitilin RB, Araújo MS, Buschini MLT. Individual specialization in the hunting-wasp *Trypoxylon* (*Trypargilum*) agamemnon Richards (Hymenoptera: Crabronidae). Zool Stud. 2012; 51: 655–662. Available from: http://zoolstud.sinica.edu.tw/Journals/51.5/655.pdf

[20] Santoro D, Polidori C, Asís JD, Tormos J. Complex interactions between components of individual prey specialization affect mechanisms of niche variation in a grasshopper-hunting wasp. J Anim Ecol. 2011; 80: 1123–1133. 10.1111/j.1365-2656.2011.01874.x 21644980

[21] Polidori C, Mendiola P, Asís JD, Tormos J, Garcia MD, Selfa J. Predatory habits of the grasshopper-hunting wasp *Stizus continuus* (Hymenoptera: Crabronidae): diet preference, predator-prey size relationships and foraging capacity. J Nat Hist. 2009; 43: 2985–3000.

[22] Bolnick DI, Svanbäck R, Fordyce JA, Yang LH, Davis JM, Hulse CD, Forister ML. The ecology of individuals: incidence and implications of individual specialization. Am Nat. 2003; 161: 1–28. 10.1086/343878 12650459

[23] Costa-Pereira R, Rudolf VHW, Souza FL, Araújo MS. Drivers of individual niche variation in coexisting species. J Anim Ecol. 2018; 87: 1452–1464. 10.1111/1365-2656.12879 29938791

[24] Bohart RM, Menke AS. Sphecid wasps of the world: a generic revision. Berkeley: University of California Press; 1976.

[25] Krombein KV. Trap-nesting wasps and bees: Life stories, nests and associates. Washington D.C.: Smithsonian Press; 1967.

[26] Coville RE. Wasps of the genus *Trypoxylon* subgenus *Trypargilum* in North America (Hymenoptera: Sphecidae). Univ Cal Publ Entomol. 1982; 97: 1–147.

[27] Blackledge TA, Coddington JA, Gillespie RG. Are three-dimensional spider webs defensive adaptations? Ecol Lett, 2003; 6: 13–18. 10.1046/j.1461-0248.2003.00384.x

[28] Cardoso P, Pékar S, Jocqué R, Coddington JA. Global patterns of guild composition and functional diversity of spiders. PLoS ONE. 2011; 6: e21710 10.1371/journal.pone.0021710 21738772 PMC3126856

[29] Sanders D, Vogel E, Knop E. Individual and species-specific traits explain niche size and functional role in spiders as generalist predators. J Anim Ecol. 2015; 84: 134–142. 10.1111/1365-2656.12271 25041766

[30] Traveset A, Tur C, Trøjelsgaard K, Heleno R, Castro-Urgal R, Olesen JM. Global patterns of mainland and insular pollination networks. Glob Ecol Biogeogr. 2016; 25: 880–890. 10.1111/geb.12362

[31] Grant PR. Evolution on islands. Oxford: Oxford University Press; 1998.

[32] Drake DR, Mulder CPH, Towns DR, Daugherty CH. The biology of insularity: an introduction. J Biogeogr. 2002; 29: 563–569. 10.1046/j.1365-2699.2002.00706.x

[33] Rodríguez-Estrella R. Los oasis de Baja California Sur: su importancia y conservación In: Rodríguez-Estrella R, Cariño Olvera M, Aceves García F, editors. Reunión de análisis de los oasis de Baja California Sur: importancia y conservación. La Paz, BCS: CIBNOR, UABCS, SEMARNAT; 2004 p. 1–8.

[34] Rodríguez-Estrella R, Arriaga L. Implicaciones ecológicas de las actividades humanas en la biota asociada a los oasis In: Arriaga L, Rodríguez-Estrella R, editors. Los oasis de la península de Baja California. La Paz, BCS: CIBNOR; 1997 p. 285–292.

[35] Jiménez ML, Nieto-Castañeda IG, Correa-Ramírez MM, Palacios-Cardiel C. Las arañas de los oasis de la región meridional de la península de Baja California, México. Revista Mexicana de Biodiversidad, 2015; 86: 319–331. 10.1016/j.rmb.2015.04.028

[36] MacArthur RH. The theory of the niche In: Lewontin RC, editor. Population biology and evolution. New York, NY: Syracuse University Press; 1968 p. 159–176.

[37] Wiggins IL. Flora of Baja California. Stanford, CA: Stanford University Press; 1980.

[38] Servicio Meteorológico Nacional (SMN); 2017. [cited 2018 April 10]. Available from: http://smn.cna.gob.mx/es/.

[39] INEGI. Climate maps and datasets; 2017. [cited 2017 June 16] Available from: www.inegi.org.mx

[40] Uetz GW, Halaj J, Cady AB. Guild structure of spiders in major crops. J Arachnol. 1999; 27: 270–280.

[41] Chao A, Chiu CH, Jost L. Unifying species diversity, phylogenetic diversity, functional diversity, and related similarity and differentiation measures through Hill numbers. Annu. Rev. Ecol. Evol. Syst. 2014; 45: 297–324. 10.1146/annurev-ecolsys-120213-091540

[42] Colwell RK, Coddington JA. Estimating terrestrial biodiversity through extrapolation. Philos Trans R Soc Lond B Biol Sci. 1994; 345: 101–118. 10.1098/rstb.1994.0091 7972351

[43] Hsieh TC, Ma KH, Chao A. (2018) iNEXT: iNterpolation and EXTrapolation for species diversity. R package version 2.0.17. ed2018

[44] R Core Team. R: A language and environment for statistical computing. 3.6.1 2019-07-05 ed. Vienna, Austria 2019.

[45] Anderson MJ. A new method for non-parametric multivariate analysis of variance. Austral Ecol. 2001; 26: 32–46. 10.1111/j.1442-9993.2001.01070.pp.x

[46] Anderson MJ, Ellingsen KE, McArdle BH. Multivariate dispersion as a measure of beta diversity. Ecol Lett. 2006; 9: 683–693. 10.1111/j.1461-0248.2006.00926.x 16706913

[47] Kruskal JB, Wish M. Multidimensional scaling. California: Sage Publications; 1978.

[48] Oksanen J, Blanchet FG, Friendly M, Kindt R, Legendre P, McGlinn D, et al. vegan: Community Ecology Package. R package version 2.5–3. ed2018.

[49] Bates D, Maechler M, Bolker B, Walker S. Fitting Linear Mixed-Effects Models Using lme4. J Stat Softw. 2015; 67: 1–48.

[50] Colwell RK, Futuyma DJ. On the measurement of niche breadth and overlap. Ecology. 1971; 52: 567–576. 10.2307/1934144 28973805

[51] Hulbert SH. The measurement of niche overlap and some relatives. Ecology. 1978; 59: 67–77.

[52] Levins R. Evolution in changing environments: Some theoretical explorations. Princeton: Princeton University Press; 1968.

[53] Zhang J. spaa: SPecies Association Analysis. R package version 0.2.2. ed2018.

[54] Blüthgen N, Menzel F, Blüthgen N. Measuring specialization in species interaction networks. BMC Ecol. 2006; 6: id9 10.1186/1472-6785-6-9 PMC1570337 16907983

[55] Blüthgen N, Menzel F, Hovestadt T, Fiala B, Blüthgen N. Specialization, constraints, and conflicting interests in mutualistic networks. Curr Biol. 2007; 17: 341–346. 10.1016/j.cub.2006.12.039 17275300

[56] Blüthgen N, Fründ J, Vázquez DP, Menzel F. What do interaction network metrics tell us about specialization and biological traits? Ecology. 2008; 89: 3387–3399. 10.1890/07-2121.1 19137945

[57] Dormann CF, Gruber B, Fruend J. Introducing the bipartite Package: Analysing Ecological Networks. R news. 2008; Vol 8/2, 8–11.

[58] Dormann CF, Fruend J, Bluethgen N, Gruber B. Indices, graphs and null models: analyzing bipartite ecological networks. Open Ecol J. 2009; 2: 7–24. 10.2174/1874213000902010007

[59] Evans HE. A revision of the Mexican and Central American spider wasps of the subfamily Pompilinae (Hymenoptera: Pompilidae). Mem Am Entomol Soc. 1966; 20: 1–442.

[60] Izenman AJ. Modern multivariate statistical techniques. Philadelphia: Springer; 2013.

[61] Revelle W. psych: Procedures for Personality and Psychological Research. R package version 1.8.10. ed2018.

[62] Novosolov M, Rodda GH, Gainsbury AM, Meiri S. Dietary niche variation and its relationship to lizard population density. J Anim Ecol. 2018; 87: 285–292. 10.1111/1365-2656.12762 28944457

[63] Jiménez ML, Tejas A. Las arañas presa de la avispa lodera Trypoxylon (Trypargilum) tridentatum tridentatum en Baja California Sur, México. Southwestern Entomologist, 1994; 19: 173–180.

[64] Domínguez K, Jiménez ML. Composition of spider prey captured by the wasp Trypoxylon (Trypargilum) tridentatum tridentatum in two habitats in an oasis in Baja California Sur, México. Can Entomol. 2008; 140: 388–392. 10.4039/n07-048

[65] Llinas-Gutiérrez J, Jiménez ML. Arañas de humedales del sur de Baja California, México. An Inst Biol. 2004; 75: 283–302. Available from: http://www.redalyc.org/articulo.oa?id=45875205

[66] Piel WH. The systematics of Neotropical orb-weaving spiders in the genus *Metepeira* (Araneae: Araneidae). Bull Mus Comp Zool. 2001; 157: 1–92.

[67] Blackledge TA, Wenzel JW. Silk mediated defense by an orb web spider against predatory mud-dauber wasps. Behaviour. 2001; 138: 155–171.

[68] Uma DB, Weiss MR. Chemical mediation of prey recognition by spider-hunting wasps. Ethology. 2010; 116: 85–95. 10.1111/j.1439-0310.2009.01715.x

[69] Eberhard WG. The predatory behaviour of two wasps, *Agenoideus humils* (Pompilidae) and *Sceliphron caementarium* (Sphecidae), on the orb weaving spider *Araneus cornatus* (Araneidae). Psyche. 1970; 77: 243–251.

[70] Rayor LS. Attack strategies of predatory wasps (Hymenoptera: Pompilidae; Sphecidae) on colonial orb web-building spiders (Araneidae: *Metepeira incrassata*). J. Kans. Entomol. Soc. 1996; 69: 67–75.

[71] Punzo F, Ludwig L. Behavioral responses to *Pepsis thisbe* (Hymenoptera: Pompilidae) to chemosensory cues associated with host spiders. J Insect Behav. 2005; 18: 757–766. 10.1007/s10905-005-8738-0

[72] Punzo F. Female spider wasps, *Anoplius splendens* Driesbach (Hymenoptera: Pompilidae), learn to associate the odor of host feces with the presence of the host. J Entomol Sci. 2006; 41: 202–210. 10.18474/0749-8004-41.3.202

[73] Foelix RF. Biology of spiders. New York: Oxford University Press; 2011.

[74] Blackledge TA, Pickett KM. Predatory interactions between mud-dauber wasps (Hymenoptera, Sphecidae) and Argiope (Araneae, Araneidae) in captivity. J Arachnol. 2000; 28: 211–216. 10.1636/0161-8202(2000)028[0211:PIBMDW]2.0.CO;2

[75] Machovsky-Capuska GE, Miller MGR, Silva FRO, Amiot C, Stockin KA, Senior AM, Schuckard R, Melville D, Raubenheimer D. The nutritional nexus: Linking niche, habitat variability and prey composition in a generalist marine predator. J Anim Ecol. 2018; 87: 1286–1298. 10.1111/1365-2656.12856 29873067

[76] Judd TM, Magnus RM, Fasnacht MP. A nutritional profile of the social wasp *Polistes metricus*: Differences in nutrient levels between castes and changes within castes during the annual life cycle. J Insect Physiol. 2010; 56: 42–56. 10.1016/j.jinsphys.2009.09.002 19781547

[77] Daugherty TH, Toth AL, Robinson GE. Nutrition and division of labor: Effects on foraging and brain gene expression in the paper wasp *Polistes metricus*. Mol Ecol. 2011; 20: 5337–5347. 10.1111/j.1365-294X.2011.05344.x 22066722

[78] Cross EA, Stith MG, Bauman TR. Bionomics of the organ-pipe mud-dauber, *Trypoxylon politum* (Hymenoptera: Sphecoidea). Ann Entom Soc Am. 1975; 68: 901–916.

[79] Poisot T, Stouffer DB, Gravel D. Beyond species: why ecological interaction networks vary through space and time. Oikos. 2015; 124: 243–251. 10.1111/oik.01719

[80] Memmott J, Martinez ND, Cohen JE. Predators, parasitoids and pathogens: species richness, trophic generality and body sizes in a natural food web. J Anim Ecol. 2001; 69: 1–15. 10.1046/j.1365-2656.2000.00367.x

[81] Polidori C, Santoro D, Asís JD, Tormos J. Individual prey specialization in wasps: Predator size is a weak predictor of taxonomic niche width and niche overlap In: Polidori C. Predation in the Hymenoptera: an evolutionary perspective. Kerala: Transworld Research Network; 2011 pp. 101–121.

[82] Coello C. Effects of prey size and load carriage on the evolution of foraging strategies in wasps In: Polidori C. Predation in the Hymenoptera: an evolutionary perspective. Kerala: Transworld Research Network; 2011 pp. 23–37.

[83] Araújo MS, Gonzaga MO. Individual specialization in the hunting wasp *Trypoxylon* (*Trypargilum*) *albonigrum* (Hymenoptera, Crabronidae). Behav Ecol Sociobiol. 2007; 61: 1855–1863. 10.1007/s00265-007-0425-z

[84] Buschini MLT, Borba NA, Brescovit AD. Prey selection in the trap-nesting wasp *Trypoxylon* (*Trypargilum*) *opacum* Brèthes (Hymenprtera; Crabronidae). Braz J Biol. 2010; 70: 529–536. 10.1590/S1519-69842010000300009 20730339

[85] Buschini MLT, Borba NA, Brescovit AD. Patterns of prey selection of Trypoxylon (*Trypargilum*) *lactitarse* Saussure (Hymenoptera: Crabronidae) in southern Brazil. Braz J Biol. 2008 68: 519–528. 10.1590/S1519-69842008000300008 18833472

[86] Brändle M, Öhlschläger S, Brandl R. Range sizes in butterflies: correlation across scales. Evol Ecol Res. 2002; 4: 993–1004. Available from: http://www.evolutionary-ecology.com/issues/v04n07/ffar1430.pdf

[87] Schowalter TD. Insect ecology, an ecosystem approach. London: Academic Press; 2011.

[88] Heinrich B. Thermoregulation in endothermic insects. Science. 1974; 185: 747–756. 10.1126/science.185.4153.747 4602075

[89] Kühsel S, Brückner A, Schmelzle S, Heethoff M, Blüthgen N. Surface area–volume ratios in insects. Insect Sci. 2016; 00, 1–13. 10.1111/1744-7917.12362 27234132

[90] O'Neill KM, O'Neill RP. Thermal stress and microhabitat selection in territorial males of the digger wasp *Philanthus psyche* (Hymenoptera: Sphecidae). J Therm Biol. 1988; 13:15–20.

[91] Herrera CM. Floral biology, microclimate, and pollination by ectothermic bees in an early-blooming herb, Ecology. 1995; 76: 218–228.

[92] Heinrich B. (1993). The hot-blooded insects, strategies and mechanisms of thermoregulation. London: Springer Berlin Heidelberg 1993.

[93] Santos GMM, Presley SJ. Niche overlap and temporal activity patterns of social wasps (Hymenoptera: Vespidae) in a Brazilian cashew orchard. Sociobiology. 2010; 121–131.

[94] Kasper ML, Reeson AF, Cooper SJB, Perry KM, Austin AD. Assessment of prey overlap between a native (*Polistes humilis*) and an introduced (*Vespula germanica*) social wasp using morphology and phylogenetic analyses of 16S rDNA. Mol Ecol. 2004; 13: 2037–2048. 10.1111/j.1365-294X.2004.02193.x 15189224

[95] Morato EF, Martins RP. An overview of proximate factors affecting the nesting behavior of solitary wasps and bees (Hymenoptera: Aculeata) in preexisting cavities in wood. Neotrop Entomol. 2006; 35: 285–298. 10.1590/S1519-715566X2006000300001 18575687

